# Supplementary material for: Are carbon emissions trading and green financial instruments synergistic? -Comprehensive quantitative research based on content analysis
Source: PLoS One. 2024 Mar 7;19(3):e0298601. doi: 10.1371/journal.pone.0298601 (PMC10919601; doi:10.1371/journal.pone.0298601)
Supplement: S1 Data — (ZIP) [file pone.0298601.s001.zip › Data/地级市Z_F工作报告.zip!/╣π╢1⁄2/╓Θ║ú╩╨/└┤╫╘╣1⁄2╓┌║┼í╛┬φ┐╦╩2╛▌═°í┐_2018.docx]

各位代表：

　　我代表珠海市人民政府，向大会作政府工作报告，请予审议，并请政协委员和其他列席人员提出意见。

　　一、2017年工作回顾

　　2017年，在省委、省政府和市委的正确领导下，在市人大、市政协的监督支持下，我们深入贯彻落实党的十八大和十八届历次全会精神，认真学习贯彻党的十九大精神，以习近平新时代中国特色社会主义思想为指导，以习近平总书记对广东作出的重要指示批示精神为统领，坚持稳中求进工作总基调，落实新发展理念和以人民为中心的发展思想，团结带领全市人民携手共进、攻坚克难，较好地完成了市九届人大一次会议确定的主要目标任务，经济社会发展取得积极成效，十件民生实事全部完成，各项工作再上新台阶。

　　全市实现地区生产总值2564.73亿元、增长9.2%，增速位居全省第一；固定资产投资1662.02亿元、增长19.6%；社会消费品零售总额1128.18亿元、增长11%；规模以上工业增加值1105.62亿元、增长10.6%；实际吸收外资24.33亿美元、增长6%；外贸进出口2990.12亿元、增长8.6%；一般公共预算收入314.35亿元、增长10.4%；全体居民人均可支配收入预计为43969元、增长9.5%。

　　一年来我们主要做了以下工作：

　　（一）精准发力供给侧结构性改革，发展动能转换提速

　　“去降补”工作扎实开展。去产能取得成效，国有“僵尸企业”出清重组工作全面完成，向粤东西北地区转移落户项目26个。去杠杆稳步推进，全市规模以上工业企业资产负债率64.4%，金融机构杠杆率控制在合理水平。降成本使企业获益，全年为企业降负累计超过80亿元，规模以上工业企业利润总额369.62亿元、增长24.3%。对农村电网、地下管网、水利等十个领域的薄弱环节加大投资，全年完成补短板投资251.16亿元，完成计划的142.5%。

　　实体经济贡献率明显提升。在园区建设、招商引资、企业培育、技术改造等方面出台支持实体经济发展系列政策，启动横琴、保税、洪湾片区一体化建设，调整富山工业园管理体制，新设立智慧产业园，一批项目签约动工投产，实体经济发展活力不断增强。全市完成规模以上工业总产值4653.09亿元、增长12.9%；装备制造业增加值434.17亿元、增长13.4%；工业和建筑业对经济增长的贡献率达63.6%，比上年提高16.2个百分点。促进各类企业健康发展，产值超十亿元工业企业73家，其中超百亿元企业7家；民营经济增加值886.92亿元、增长9.1%，市属国企利润总额294亿元、增长27.5%。

　　房地产调控取得实效。实施“限购、限贷、价格备案、限售”调控措施，投机炒房得到有效遏制。全市与房地产相关的贷款增量占各项贷款增量的39.5%、比上年下降34.4个百分点，房地产投资占固定资产投资总额的40.1%、下降6个百分点，房地产业增加值占地区生产总值的7.7%、下降1.1个百分点，经济增长正逐步摆脱对房地产的过度依赖。

　　（二）着力实施创新驱动发展战略，创新环境不断优化

　　创新主体蓬勃发展。852家高新技术企业通过认定，总数突破1400家。全市完成工业技改投资194.84亿元、增长32.1%。研发机构建设加快，年主营业务收入5亿元以上工业企业实现研发机构全覆盖，规模以上工业企业研发机构覆盖率达37.5%，新增省级新型研发机构2家、省级以上创新平台90家，引进中德人工智能研究院、珠海中科先进技术研究院等项目。支持企业开展核心技术攻关，13个项目获省级以上重大科技项目立项。完善科技企业孵化育成体系，新增2家国家级、2家省级科技企业孵化器，5家国家级、8家省级众创空间，2家国家小微企业双创示范基地，1家全国“互联网+”双创示范基地，全市在孵企业超过1300家。

　　创新体系逐步完善。出台粤港澳大湾区创新高地、“双自联动”、自创区建设实施方案。预计全社会研发经费支出占地区生产总值的2.5％，PCT申请量累计达1508件。每万人口发明专利拥有量50件，居全省第二。实施人才优先引进、留学人员创业资助等措施，新引进国家“千人计划”专家43名、累计达93名，均居全省地级市首位。

　　科技金融服务能力增强，全市新增金融类企业3072家、备案创业投资企业137家、上市公司3家、“新三板”挂牌公司7家。加快知识产权强市、质量强市建设，通过国家知识产权试点城市验收，全国首个深海海洋工程装备产业知名品牌示范区成功创建。全国首批制冷设备、办公设备及耗材技术性贸易措施研究两个评议基地落地珠海。

　　创新型经济加快成长。全市高技术制造业增加值实现306.87亿元，占规模以上工业增加值的27.8%。格力智能装备制造、泰坦新动力、云洲智能等科技型企业产值翻倍增长，丽珠医药、健帆生物、赛纳打印等一批拥有核心技术的企业发展良好，纳睿达、四维科技、光驭科技等新兴企业加快成长。格力电器获第三届中国质量奖，烽火海洋等3个项目入选国家绿色制造系统集成项目。生产性服务业发展加快，新增1家国家级、3家省级工业设计中心，6家省电子商务试点企业，11家省供应链管理试点企业。促进军民融合产业发展，大型国产水陆两栖飞机AG600首飞成功。

　　（三）大力推进改革开放，发展活力进一步增强

　　改革力度持续加大。横琴自贸片区改革有新突破，新落地84项制度创新措施，9项创新措施在全省复制推广，创新跨境商事登记导办和智能办税服务模式，出台全国首部人力资本出资管理办法，搭建首个金融创新知识产权运营交易国家平台，发布首份失信商事主体联合惩戒清单。“放管服”改革继续推进，全市新承接省下放事权76项，下放市级事权44项，清理规范行政审批中介服务事项39项。建立市建设项目审批综合管理平台，56个事项实现“一门式一网式”审批。完善“互联网+政务服务”，727项行政许可事项全部进驻网厅。商事主体名称自主申报，“一照一码”登记全程电子化，“珠海易注册”开通上线，新增市场主体3.95万户、增长15.8%。27个部门合作开展诚信纳税联合激励，社会信用体系建设考核连续五年全省第一。投融资体制改革稳步推进，完成政府投资项目管理条例、建设工程招标投标管理办法修订，组建首期规模超过500亿元的珠海基金。国企改革“1+N”制度框架初步构建。

　　区域合作逐步深化。粤港澳紧密合作示范区加快建设，横琴新注册港澳企业849家、增长72%。粤澳合作产业园16个项目开工建设，粤澳中医药科技产业园注册企业达56家。粤港澳游艇“自由行”方案获批。澳门机动车出入横琴政策受惠面扩大，横琴口岸率先启动粤澳两地牌小客车检查结果参考互认。粤澳合作发展基金落户横琴，内地首家澳门银行营业性机构开业。新签署珠澳旅游、文化等多项合作协议。珠中江阳区域紧密合作取得新进展，对口帮扶阳江的37个亿元以上工业项目动工。

　　开放水平不断提升。制定开放引领发展、“一带一路”战略支点建设实施方案。成功举办中以科技创新投资大会、中拉国际博览会，中以加速器启动建设，中拉经贸合作园开园。在海关、边检、检验检疫等口岸联检单位支持下，国际贸易“单一窗口”建设加快，通关便利化水平进一步提升。外贸结构持续优化，对“一带一路”国家出口增长36.4%，高新技术产品进出口增长12.9%。全市新引进项目127个，北美、欧洲等5个驻外经贸代表处挂牌运作，招商网络进一步完善。全年接待游客总人数3980.69万人次，旅游总收入367.7亿元、增长16%。“海丝”论坛、中国国际马戏节、WTA超级精英赛、莫扎特国际青少年音乐周、留学生节等活动成功举办，珠海向世界展示了独特的魅力和形象。

　　（四）全面加快交通建设，海陆空交通体系不断完善

　　对外交通建设有新突破。港珠澳大桥主体工程全线贯通，珠海在区域的交通地位发生历史性变化，成为唯一与港澳陆桥相连的城市。珠海机场升级改造项目开工，候机楼东指廊投入使用，在飞航线69条，旅客吞吐量达921.7万人次、增长50.3%，增速位居中南地区第一。黄茅海5万吨航道开工，珠海港神华粤电煤炭码头、北港池15万吨级升级工程完工，北粮南运海铁大通道开通，高栏港货物吞吐量突破亿吨。高铁新增7条线路，通达城市47个，乘坐高铁和城际轨道出行人数达1792.87万人次、增长32%。横琴口岸及综合交通枢纽开发项目顺利推进。

　　市内路网建设加速推进。一批横跨东西、纵贯南北的交通项目加快建设，鹤港高速一期、珠峰大道、金琴快线、兴业快线北段动工建设，香海大桥、洪鹤大桥建设全面铺开，洪湾枢纽互通二期、白石桥建成通车，迎宾北路等4条主干道路沥青罩面铺设完成，诗僧路与南坦路衔接工程等一批珠中跨界道路加快实施。城际轨道市区至横琴段全线站点主体工程基本完工。

　　交通拥堵治理积极施策。全市机动车保有量超过62万辆，五年来年均增长16%，交通压力不断加大。为缓解交通拥堵，新开公交线路20条，新建成公交专用道3条，更换新能源公交车400辆。完成42个道路交叉口挖潜改造，打通断头支线道路11条，完成板樟山隧道扩容工程方案设计，建成智慧交通运行管理平台和信号协调中央控制平台。完善慢行设施，引导绿色出行，绿色公共交通出行总人次增长19.4%。

　　（五）城市建设稳步推进，生态优势持续巩固

　　新区新城建设有序铺开。横琴新区完成基础设施投资94.95亿元，马骝洲隧道、长湾隧道顺利贯通，横琴国贸大厦、洲际航运中心、励俊庞都广场等项目竣工。西部生态新城起步区完成基础设施投资53.72亿元，各片区土地清理进入收尾阶段，主干路网全面开工建设，已建成20条市政道路，25个公共服务项目和9个产城融合项目加快推进。高新区完成基础设施投资10.5亿元，一批市政设施、生态公园加快建设，高新区人民医院二期工程竣工验收。

　　城市管理出台新举措。出台城市环境清理、规范、优化、提升“1+7”工作方案，依法拆除大型户外立柱广告设施190宗，整改规范交通标志3293块，优化提升工地围挡114.4公里，修复提升市政道路和立面2101处，更换灯杆1640基，综合提升绿化节点110处，修复翻新公交亭140座。洪湾、银坑、广昌等旧村改造项目动工，乐士文化区、金湾智造大街等“工改产”项目全面启用。智慧城市建设明确了总体框架及重点领域，新增4G基站3926座、光纤接入用户数19.9万户，互联网普及率达83%，在全国智慧城市评估中排名第6。

　　生态文明建设取得新成果。获评全国首批国家生态文明建设示范市。香炉湾沙滩修复项目获评中国人居环境范例奖。完成40公里健康步道、80公里林荫道、70公里绿道、1.1万亩森林碳汇造林工程等森林城市项目建设。落实大气污染防治强化措施，淘汰黄标车1454辆，油气回收改造全部完成，加强扬尘治理，在横琴新区率先实行余泥渣土运输全密闭。建立市区镇村四级河长制，新建污水管网70公里，集中式饮用水源水质达标率100%。启动土地污染防治前期工作，开展336家重点行业企业土壤信息定性调查、393个农用地点位核实及采样。建成中信环保生物质热电一期工程。环境执法保持高压态势，开展水源保护区、“小散乱污”企业、固体废物污染防治等专项执法行动，中央环保督察交办案件全部按期办结。国内首个海洋波浪能试验项目在大万山岛启动。

　　（六）社会事业全面进步，民生保障不断加强

　　底线保障水平有新提升。九项民生支出354.14亿元、增长24.7%，占一般公共预算支出的71.7%。城镇新增就业人数46923人，城镇登记失业率控制在2.26%。低保标准提高到每人每月896元，农村五保集中和分散供养标准达到每人每年22260元和18929元，特困人员基本生活标准达到低保标准的1.6倍。为17000多名残疾人提供每人每年1920元到2640元的生活津贴。城乡居民月人均养老金提高到519元，基本医疗保险年度财政补贴提高到510元，医疗保险年度最高支付限额提高到72万元。生育保险和基本医疗保险在全国率先合并实施。超额完成省政府下达住房保障工作任务，新开工棚户区改造住房2863套，基本建成棚户区改造住房和公共租赁住房2260套。住房公积金异地转移实现“全国漫游”，缴存覆盖率全省第一。市中心粮库一期、市食品检测中心建成使用。

　　公共服务体系建设有新进步。教育事业加快发展，中大附中、中大附小、礼和小学、斗门区实验二小建成使用，珠海中学开工建设，市技师学院新校址项目顺利实施，中山大学海洋科学学院楼、多学科交叉平台楼等基建项目启动，北师大珠海分校向北师大珠海校区转型。医疗卫生事业均衡发展，狠抓市妇幼保健院异地新建、市慢性病防治中心等项目进度，市精神卫生专科住院部建成使用，金湾中心医院正式运营，翠香、湾仔等社区养老服务中心投入使用。医药体制改革深化，全面取消公立医院药品加成，在省内率先推进药品和医用耗材配送改革。文体事业繁荣发展，建成7个市民艺术中心、12个社区体育公园、316家“数字农家书屋”，全国帆船帆板锦标赛、广东旅游文化节、广东群众音乐舞蹈花会、南粤古驿道骑行、国内首届市级应急救援运动会等活动成功举办。引导社会组织有序参与社会治理，社会福利与慈善事业加快发展。平安珠海建设深入推进，110违法犯罪警情下降4.1%。社会大局持续稳定，未发生重特大生产安全事故。

　　新农村建设有新成效。落实农村人居环境综合整治三年行动计划，完成一批路灯照明、污水处理、农田水利、厕所改造等设施建设。发展特色生态农业，白蕉海鲈获评中国百强农产品区域公用品牌，岭南大地度假区获批全国首批田园综合体试点，斗门镇获评全国特色小镇，斗门区获评全国休闲农业和乡村旅游示范区。对4131户、7402人的农村低收入群体开展精准帮扶，启动572户住房改善工作。完成农村土地承包经营权确权登记颁证任务。

　　脱贫攻坚有力推进。认真落实中央和省交办的扶贫任务。精准扶贫阳江市、茂名市211个贫困村，建设特色产业基地133个。开展对口云南怒江州扶贫协作项目30个。积极实施对口支援四川甘孜州稻城县和理塘县、西藏林芝市米林县及米林农场、重庆巫山县等藏区库区工作。

　　一年来，我们进一步加强政府自身建设，改进机关作风，提升行政效能，激励广大干部勇于担当、积极作为。持续推进廉洁政府建设，落实政府部门党组（党委）主体责任，强化廉政风险防控，加强审计结果运用。推进法治政府建设，提请市人大审议法规草案2部、制定政府规章6部，实现政府法律顾问聘任全覆盖，获评依法行政全省优秀城市。依法接受市人大及其常委会的监督，自觉接受市政协的民主监督，主动接受社会和舆论监督，认真办理市人大建议119件和市政协提案359件。双拥、民族、宗教、对台、侨务、统计、人防、气象、档案、仲裁、地方志、司法行政、政务公开、海防打私等工作取得新成效。

　　各位代表，2017年8月，强台风“天鸽”正面袭击珠海，面对有气象记录以来最大的台风灾害，在省委、省政府和市委的坚强领导下，全市人民万众一心、守望相助、不畏艰难、顽强奋战，迅速恢复了生产生活秩序，充分展现了珠海人民的文明素质，体现了大家对这座城市的热爱、对政府工作的大力支持。过去一年的成绩来之不易，这是全市人民共同奋斗的结果。我代表市政府，向为珠海建设发展付出辛勤劳动的全市人民，致以崇高的敬意！向给予政府工作大力支持的人大代表和政协委员，向各民主党派、工商联、无党派人士和人民团体，向关心支持珠海发展的中央和省驻珠单位、驻珠部队、省直部门、兄弟城市、港澳台同胞和国际友人表示衷心的感谢！

　　同时，我们清醒地看到，相对于广大市民日益增长的美好生活需要，珠海市发展不平衡不充分的矛盾还比较突出，政府工作还存在不少问题和不足。一是实体经济基础比较薄弱，产业龙头项目偏少、掌握核心技术的企业偏少，产业结构调整和新动能培育任务艰巨。二是东西之间、城乡之间、陆海之间发展差距较大，城市框架尚未完全拉开，西部地区和海岛公共配套建设相对滞后，建成区旧厂房、城中村、老旧社区等有待改造提升。三是社会民生事业存在短板，公办优质学位相对不足、中心城区交通拥堵、城市精细化管理水平不高等群众强烈关注的问题，还未能有效解决。四是特区开拓创新的锐气有所弱化，部分干部存在“小富即安”的心态，少数干部存在精神懈怠和不担当、不作为现象。对此，我们一定要保持清醒头脑，增强忧患意识和进取意识，采取有效措施，切实加快解决。

　　二、2018年工作部署

　　2018年是全面贯彻落实党的十九大精神的开局之年。党的十九大作出了中国特色社会主义进入新时代的重大判断，描绘了决胜全面建成小康社会、全面建设社会主义现代化国家的宏伟蓝图，为谱写新时代珠海改革发展新篇章指明了方向。随着“一带一路”、大湾区、自贸区、自创区建设等国家政策优势交汇叠加，特别是港珠澳大桥建成通车，珠海历史性地站在了粤港澳大湾区的中心连接点，作为广东省副中心和珠江西岸核心城市，珠海的战略地位更加凸显。我国经济发展进入新时代，珠海建市以来形成的生态环境、产业品质、城市格局、市场环境等优势，为推动高质量发展奠定了坚实基础。当前，国家和省对珠海寄予厚望，外界普遍看好珠海发展，全市人民对未来充满期待。面对十九大提出的新使命新任务，大桥通车后的新机遇新挑战，人民群众对美好生活的新期待新要求，政府工作要有新气象，更要有新作为。要结合“大学习、深调研、真落实”活动，更加自觉地落实中央、省和市委的决策部署，更加准确地认识和把握市情，更加主动地谋划和推动改革发展。各项工作都要坚持高标准，跟最好的比，向最好的学，为人民群众创造更有品质的生活。

　　做好今年政府工作的总体思路是：全面贯彻党的十九大和中央经济工作会议、中央农村工作会议精神，以习近平新时代中国特色社会主义思想为指导，按照“三个定位、两个率先”和“四个坚持、三个支撑、两个走在前列”的要求，贯彻落实省委十二届二次、三次全会和市委八届三次、四次全会精神，坚持稳中求进工作总基调，坚持新发展理念和以人民为中心的发展思想，贯彻高质量发展的要求，深入实施创新驱动和开放引领“两大战略”，统筹推进稳增长、促改革、调结构、惠民生、防风险各项工作，更加注重强化在大湾区中的交通枢纽功能，更加注重发挥横琴和西部生态新区的发展潜力，更加注重增强人民群众的获得感，进一步提升经济质量和城市品质，以优异成绩迎接改革开放40周年。

　　建议2018年经济社会发展的预期目标和约束性指标为：地区生产总值增长8.5%左右；规模以上工业增加值增长10.5%；社会消费品零售总额增长11%；固定资产投资增长18%；外贸进出口保持正增长；一般公共预算收入增长9%；居民人均可支配收入增长9%；居民消费价格涨幅、城镇登记失业率均控制在3%以内；节能减排降碳和大气、水环境质量完成省下达的目标任务。

　　围绕上述发展目标，重点安排以下工作：

　　（一）深化供给侧结构性改革，进一步提升经济发展质量

　　推进“破、立、降”各项工作。破除无效供给，巩固国有“僵尸企业”出清成果，推动非国有“僵尸企业”年底前实现市场出清。着力培育新动能，出台实施现代产业体系规划。大力降成本，全年为企业减负60亿元。坚持精明增长，清理闲置用地，督促供地两年以上的36个未开工项目明确开工时间，推动近两年供地的160个工业项目如期动工。提升产业存量，继续开展“十百千”工业企业梯度培育。启动新一轮技改三年行动计划，推动260家规模以上工业企业实施技术改造。做大产业增量，加强招商引资工作，完善商务、规划、国土等部门联动的招商机制，更加注重引进项目的动工达产时限和产值税收贡献。

　　扩大投资、消费、外贸需求。加大投资力度，安排年度重点项目411项、投资780亿元，力促64个项目建成投产、149个项目开工建设，带动全社会固定资产投资超过2000亿元。加强政府与社会资本合作，运用好珠海基金。扩大消费需求，推动流通追溯体系建设，构建三级城市配送体系，申报国家跨境电子商务综合试验区。加强城市宣传，加快建设滨海国际休闲旅游目的地和国家全域旅游示范区，推动珠海旅游产品品牌设计，发挥横琴长隆等龙头项目作用，带动旅游消费。推动外贸持续增长，扶持贸易平台建设，发展外贸产业集群，推进保税仓储业务扩大进出口，积极发展外贸综合服务平台、市场采购贸易出口等外贸新业态。

　　着力发展实体经济。切实做好系列支持政策的宣讲、落实、评估和完善，加快扶持资金审批拨付，强化金融服务支撑。划定产业保护区，保持产业用地足够规模，把好地留给好项目。推进园区扩能增效，全年园区建设投入不低于90亿元。出台实施先进装备制造业三年行动计划，申报“中国制造2025”国家级示范区，推动伟创力工业4.0制造基地、中海福陆海洋工程装备三期等一批项目动工，加快运泰利智能产业园、恩捷电池隔膜等项目建设。深入实施“互联网+先进制造业”，发展工业互联网。推动民营经济发展，推行政企云微门户，组建中小企业先进技术研究院。弘扬劳模精神和工匠精神，开展质量提升行动，推进国家新能源汽车动力电池及电驱动系统质检中心、省质量监督海洋工程装备检验站等项目建设，建成现代办公设备和打印耗材知名品牌示范区。支持富山工业园、智慧产业园、航空产业园等创建国防科工军民融合创新示范基地。办好2018中国集成电路设计年会。

　　促进房地产市场平稳健康发展。严格落实“房子是用来住的，不是用来炒的”总体定位，保持房地产市场调控政策连续性和稳定性，坚决遏制投机炒房。优化人才购房政策措施，更好地满足人才在珠海创业生活的居住需求。鼓励住房租赁市场发展，加快建立多主体供应、多渠道保障、租购并举的住房制度。

　　（二）加快创新型城市建设，打造粤港澳大湾区创新高地

　　完善创新创业环境。制定推动创新驱动发展再提速行动计划，出台支持企业创新能力提升、科技成果转化、鼓励科技创新公共服务和资源共享等系列政策，修订创新相关扶持资金管理办法，提高政策的精准度和有效性，营造更加有利于创新要素集聚的环境。优化人才政策体系，加快实施人才卡、高层次人才“认定+评审+举荐”等人才新政，全年引进省创新创业团队不少于3家、国家“千人计划”专家不少于30名、各类人才不少于2万名。制定人才安居办法，加大人才公寓和共有产权房建设，着力解决人才尤其是青年人才对住房的后顾之忧。探索投贷结合、投债联动、投担联动等科技金融服务模式，发挥财政资金和珠海基金引导作用，发展天使投资、创业投资，新增备案创业投资机构70家。开展标准国际化创新型城市试点、国家知识产权示范城市创建工作。

　　加强创新载体建设。实施高新技术企业树标提质行动，力争企业数达1550家。瞄准国内外最具成长潜力、最具投资价值、最具创新力企业，开展精准招商。对接国家和省重大科技专项，组织实施一批市级重大技术攻关项目。扶持和培育新型研发机构，争取新增省级新型研发机构2家、省级以上创新平台50家。落实孵化器建设规划，建设大湾区博士和博士后（珠海）创新创业孵化基地。推动创新型应用型高校建设，加快中山大学珠海校区基础设施项目建设，力争北京师范大学珠海校区秋季开始招生。完善“菁创荟”服务平台，支持青年创新创业。对接广深科技创新走廊建设，承接科技项目和研发成果转移。

　　落实“双自联动”方案。支持高新区企业利用横琴保税融资租赁政策和与生产有关的进口免税政策，进口高价值科研设备。支持横琴企业利用高新区科技型企业集聚优势，开展研发设计、检测维修等保税业务。发挥创新主平台作用，主动融入国内外科技创新网络，推进粤澳合作产业园、中以加速器、中德人工智能研究院等项目建设。完善联动发展机制，打造横琴科学城、横琴国际科创中心等联动发展载体。

　　（三）深入推进重点领域改革，提升开放合作水平

　　打造横琴高水平对外开放门户枢纽。探索自贸区更大改革自主权，在口岸监管模式创新等方面争取更大突破。优化法治环境，探索建立粤港澳大湾区商标知识产权跨境合作机制，支持横琴国际知识产权交易中心发展。推进国家人才管理改革试验区建设，完善创业团队激励、人才引进等政策。发挥横琴辐射作用，带动保税区、洪湾片区功能升级，加强横琴与各区招商联动。促进与港澳更深层次合作，推动粤澳中医药科技产业园、创新方等合作项目首期建成运营。建成深井二线通道。加大对港澳创业项目的扶持，培育不少于200个澳门创业项目。推进珠港澳旅游合作，推动游客资源共享。配合澳门世界休闲旅游中心建设，创建横琴国际休闲旅游岛，争取横琴、澳门之间人员往来更加便利的措施。

　　推动改革举措落地生效。深化“放管服”改革，做好省政府下放事项的有效承接，完善市级事权下放的后续监管和动态调整机制。推动人往基层走、钱向基层投、政策朝基层倾斜，充分发挥区级积极性。加强市级在国土、规划、重大项目布局上的统筹。深化城市管理机制改革，做实网格化管理，确保每一寸土地、每一个角落都有人负责。推进政务服务标准化，启动“减证便民”工作。将全部市级事项纳入市建设项目审批综合管理平台，进一步优化并联审批流程。落实“证照分离”，提高商事主体开办便利度。加强社会信用体系建设，积极创建国家信用示范市。扎实推进国企改革，提升国企竞争力。完善公共设施建设模式，更加注重城市运营，土地出让时应充分考虑教育、卫生、文化、体育、养老、社区服务等设施的配建。

　　构建大湾区时代开放合作新格局。抢抓港珠澳大桥建成通车机遇，推动珠港澳物流合作园建设，打造大桥经济区。推动珠港澳三地机场合作，积极争取珠海机场开通国际航线和设立保税物流中心（B型）。加快高栏港综合保税区申报建设，拓展珠海港国际航运服务体系，打通“川贵广—南亚”国际物流大通道。以欧美和“一带一路”沿线为重点，发挥驻境外经贸代表处作用，加强经贸交流合作。跟踪落实中以科技创新大会和中拉国际博览会成果，推动中以产业园、以色列中国（珠海）离岸创新中心建设，发挥中拉经贸合作园平台作用，承办好中拉企业家峰会。高质量办好第十二届中国国际航空航天博览会。推动会展中心二期建设，发挥国有场馆优势，引进培育1—2个国际精品专业会展品牌。做好珠海台创园转型升级，推进二期建设。深化珠中江阳合作，做好对口阳江产业帮扶工作。完善与黑龙江黑河市对口合作机制，加强农业、经贸、旅游等领域合作。

　　（四）推动交通内畅外联，建设区域交通枢纽城市

　　打好交通建设提速战。重点交通项目建设要快马加鞭。要畅通对外交通，加快完善港珠澳大桥配套设施及衔接路网建设，积极推进金琴快线北延段等项目对接深中通道，配合推进珠江肇高铁前期工作，争取将深珠高铁纳入国家和省相关规划，推动开展黄茅海大桥前期工作。完善城市组团交通网络，继续开展地铁前期和相关工作，加快东西通道香海大桥、洪鹤大桥、鹤港高速一期和南北通道兴业快线北段、金琴快线、情侣路南段主辅线工程建设。加快十字门隧道、黑白面将军山隧道、大横琴山隧道建设。一季度开工金海大桥，上半年开工板樟山新增隧道、香海大桥支线，年内开工古元大道。

　　加强港口、机场、口岸建设。开展珠海机场总体规划修编，推进机场改扩建和机场综合交通枢纽工程，加快机场升级改造和航空物流园建设进度。加速发展民航客货运输，增开航线航班。完成通用机场一期建设。增强港口集疏运功能，发展壮大西江港口联盟，推进高栏集装箱码头二期等项目建设，推动北方石油石化码头等项目开工，启动高栏港区20万吨级主航道前期工作。推进洪湾渔港建设，打造国际渔业物流港。推进横琴口岸综合交通枢纽和青茂口岸建设、湾仔口岸迁建、拱北口岸和九洲港口岸改造，加强智慧海防与打私建设。

　　综合施策治理交通拥堵。加强交通组织，做到哪里有拥堵，哪里就有交警引导。强化道路挖潜，打通断头路，建设下穿车道和美观实用的人行天桥。落实公交优先，加大车辆投放，科学安排公交线路、班次和密度，提高公交准点率和舒适度。优化道路施工方案，一定要减少对市民出行影响。规范共享单车管理，加快非机动车道优化改造。发展智慧交通，规范路内停车。鼓励建设机械式立体停车设施。

　　（五）全面实施乡村振兴战略，着力解决区域发展不平衡不充分问题

　　做好新时代“三农”工作。推进农业供给侧结构性改革，支持斗门打造供港供澳绿色食品示范区。完善支持农村一二三产业融合发展的配套措施，打造一批生态休闲农庄示范点，加快田园综合体试点项目建设。打造“互联网+现代农业”新模式，发展专业合作社、农业综合体、家庭农场等业态，完善村级电商服务网络。建设高标准基本农田3.34万亩，启动粮食生产功能区和重要农产品保护区划定工作。继续加大对农业龙头企业扶持力度，加强农业生产基地监管，提高农产品质量水平。探索推出更多涉农保险，提高农业抗灾能力。推进美丽宜居乡村建设，落实农村人居环境整治计划。加强水利基础设施建设，加快农村农田低压电网改造，提升乡村道路综合管养标准。抓好农村集体产权制度改革试点，完成农村地籍调查建库。健全市内精准帮扶长效机制，着力解决低收入群体生产生活困难。坚决打赢脱贫攻坚战，继续推进对阳江市、茂名市贫困村的精准帮扶工作，扎实做好对口云南怒江州东西部扶贫协作和对口支援藏区库区工作。

　　加快西部生态新区建设。高标准建设金湾、斗门、富山和平沙四大片区，推动产城融合。加快建设鸡啼门特大桥、双湖路、平华大道、富山新城大道、金湾体育中心、黄杨河湿地公园、平沙新城中心公园等项目，建成双湖路中段、航空城三期市政道路、金湾一中配套道路等项目。推动优质公共服务资源向西部地区倾斜，上半年开工建设西部医疗中心，推动珠海中学尽快建成。

　　促进海岛地区开发建设。海洋海岛资源是珠海发展的优势所在，也是潜力所在。要落实好海洋督查问题整改，强化围填海综合管控能力，在加强海洋保护中适度开发利用海洋资源。完善海岛基础设施，加快东澳岛客运码头、大万山岛万山湾客运码头、桂山岛十三湾防波堤建设，启动外伶仃石涌湾码头、唐家港陆岛交通补给码头项目建设。推进桂山岛海上风电、万山区海岛新能源微电网示范项目建设，改造扩容外伶仃、大万山岛水库，支持海岛路网建设改造。加密轮渡班次，改善轮渡服务。推动粤港澳游艇“自由行”政策落地，推进三角岛运动休闲与科教示范项目建设，提升海洋海岛旅游品质，加快海洋经济发展步伐。全面开展入海排污口整治，实现海岛存量垃圾打包外运、主岛污水无害化处理全覆盖，建成东澳岛和大万山岛污水无害化处理工程。设立海洋生态修复和生态补偿专项资金。继续开展蓝色海湾和海洋牧场建设。

　　（六）深化生态文明建设，打造美丽中国样板城市

　　巩固提升生态优势。牢固树立社会主义生态文明观，提供更多优质生态产品，新建5个市政特色公园和一批社区公园、20公里健康步道、30公里城市绿道，改造提升40公里林荫道。加强淇澳岛生态保护，完善珠海古驿道建设。加快推进国家海绵城市试点建设，新开工建设一批综合管廊。完成全国水生态文明城市建设试点工作。深化水、大气和土壤污染综合治理。全面推进“河长制”三年行动计划，加大黑臭水体和江河水质整治。新建污水管网40公里，新增污水处理能力10万吨/日，启动污水管网改造，推进香洲污水处理厂三期扩建和拱北、吉大、南区污水处理厂提标改造工程，完成三灶、平沙污水处理厂提标改造及扩建工程。打好蓝天保卫战，落实扬尘管理和机动车污染控制，上半年在全市推开余泥渣土运输全密闭。加强土壤污染防治，推进土壤污染详查。加大环境执法力度，切实把污染源管住。继续推进生活垃圾分类工作。推动中信生态环保产业园建设，加快环保生物质热电二期、污泥处置中心一期、餐厨垃圾处理、医疗废物处理等项目进度，力争率先在全省实现原生垃圾零填埋。

　　提高城市现代化水平。珠海环境优美，具备打造样板城市的底气和条件。要对标国内外先进城市，高标准推进规划建设管理，让珠海更具现代化气息，更加宜居宜业宜游，市民更有归属感、幸福感、自豪感。开展新一轮城市总规编制，推进“东接西拓南进北联”，尽快拉开城市格局，实现生产空间集约高效、生活空间宜居适度、生态空间山清水秀。加强城市设计，注重功能使用、景观效果、文化特色及建筑平面、立面、天面的协调统筹。加快横琴、保税、洪湾片区一体化建设，打造开发强度适中、产业聚集高端、生态环境优美，与澳门交相辉映的珠海城市新中心，高标准建设十字门中央商务区，推进马骝洲“一河两岸”规划建设。推动唐家湾科教新城建设，推进北围、后环、前环等片区路网建设，加快布局优质公共服务资源。

　　提高城市管理精细化水平。进一步明确职责、细化标准、强化考核，建立常态化机制，既重建设，更重管理，像绣花一样精细管理城市，营造更加干净、整齐、平安、优美的环境。开展主干道和重点区域环境综合提升，统筹考虑天际线、道路桥梁、建筑立面、园林绿化、广告招牌、灯光照明等城市风貌。完善主城区主要道路沥青罩面铺设，完善大中型桥梁景观亮化，优化提升公园、绿地，打造城市阳台等一批山水相连、亲山近水的标志项目。加强项目招投标、设计、施工、监理、验收等全流程管理，努力做到造价合理、质量提高、施工安全、工期缩短、扬尘噪音减少。推行标准化施工围挡，设置明显标牌，让市民知晓项目情况。继续开展违法建设专项治理。及时修补破损路面、护栏等市政设施，启动“厕所革命”新三年行动计划。加强环卫基础配套保障，提高保洁人员待遇和机械化水平。进一步提升绿化水平，科学选择树种、把握种植密度、精细修剪管养，形成高低错落、疏密有度、色彩丰富的景观效果。推进新型智慧城市建设，实施大数据发展战略，打造全市政务信息枢纽，建设“数字政府”。加强与通讯运营商和行业龙头企业的战略合作，推进大数据中心等第一批20个项目建设，推动智慧园区服务平台等第二、第三批25个项目启动。加快推进4K电视应用。

　　积极稳妥推进“三旧”改造。完善“三旧”改造政策措施，理顺改造审批环节，让老城区焕发新活力。统筹兼顾公共利益和土地权利人、投资主体的利益，重点推进安全隐患突出、公共配套缺乏、功能用途已不符合城市发展规划的区域改造。加强规划管控，保护城市肌理，采用连片改造、小拆小建和修旧如旧等模式，分类推进改造项目。旧工业区改造鼓励“工改工”“工改产”，严格限制“工改居”，重点推进“三溪”产业小镇、丽珠旧厂房改造等项目建设。推动东桥、永丰、上冲、翠微等旧村启动改造工作，优先推进村民改造意愿一致的项目。继续推进拱北口岸地区改造项目。

　　加强安全应对能力建设。落实安全生产责任，深化风险排查管控，坚决遏制重特大安全事故。狠抓火灾高风险区域专项整治，建立森林防火“4+1”体系。推进消防设施增量提质，升级和建成一批小型、微型消防站，开展畅通消防通道和逃生通道专项行动。加强城中村综合治理，完善出租屋管理系统。加强校园安全建设，提升中小学校、幼儿园、托管中心安全管理水平。加快食品集中加工区、农贸市场升级改造等硬件建设，完成省食品安全示范试点城市创建工作。实现瓶装液化气供应全过程电子化监管和全电子化服务。加强三防能力建设，实施海堤提升工程，加强地质、内涝等灾害防治。落实基础设施人防要求。做好公共安全风险普查分析及应急预案，推广无人机、大数据等技术运用，完善海陆空立体应急救援体系。推动全市公共安全教育场所建设，提高群众自救互救技能。加快完善社会治安防控体系，全面提升“主动预警、快速反应”核心战斗能力，推动全民禁毒工程，完善反恐责任体系，严厉打击违法犯罪活动，让各类犯罪分子无所遁形。加强地方金融风险防范，妥善果断处理金融违法活动。加强和创新社会治理，促进社会组织健康有序发展，预防和化解社会矛盾。

　　（七）提高民生保障水平，增强人民群众获得感

　　加强就业服务和社会保障。实施就业优先战略，组织公共实训5000人次，实施在岗职工技能提升培训1万人次，城镇新增就业4万人，推行就业服务全城通办，启动人力资源服务产业园建设。出台就业困难人员认定和就业援助办法，建设高技能人才公共实训基地和高技能人才培养示范基地。保障好底线民生，加大对低保对象、特困人员、受灾群众等群体的帮扶救助力度。大力发展养老事业，落实民办养老机构资助办法，建立居家养老服务信息平台，深化医养结合试点工作，推动市级养老机构、社区居家养老服务站建设。建立健全心理健康服务体系。为全市户籍残疾人每人每年购买意外伤害保险100元。加强住房保障信息化建设，进一步扩大住房保障覆盖面。实施工伤保险浮动费率办法，开展失业保险浮动费率试点，进一步降低企业社保缴费成本。实施医疗保险病种点数法支付，降低基本医疗保险一档费率。

　　大力发展教育和医疗卫生事业。深入落实中小学办学联盟工作，促进东西部义务教育优质均衡发展。推进白藤东小学、北山小学、横琴一中、凤凰中学、梅华中学、珠峰实验学校等项目建设，增加优质学位供给。完善中高职衔接培养机制，加快市技师学院建设，推动金湾校区秋季招生。落实特殊教育提升计划，加快自闭症支持体系建设。支持民办教育发展，加强民办学校分类管理。推进市人民医院科研综合楼、中大五院外科大楼、市第二中医院、市第五人民医院、横琴新区医院等项目建设，加快高层次卫生人才队伍建设，集中力量打造区域优势学科专科群，全力支持市人民医院、中大五院进军全省“30强”医院。推进健康服务联合体、专科区域联盟建设。做实家庭医生签约服务，重点覆盖困难家庭、慢性病家庭、老人家庭。扩大妇女“两癌”免费检查覆盖范围。做好优生优育工作。完善社区卫生服务，落实分级诊疗制度。

　　健全公共文化服务体系。建立文明城市创建长效机制，持续提升市民文明素质。加强文化遗产保护传承，实施共乐园、山房路、会同村共同改造提升，完成拉塔石炮台遗址优化提升，推进容闳故居遗址修复一期工程，提升和扩展杨匏安陈列馆，加强苏兆征、林伟民等革命先驱及陈芳、唐国安等名人故居和陈列馆的保护利用。全面推进市区镇村四级文化设施达标，启动国家公共文化服务体系示范项目创建。推动市博物馆和规划展览馆年内对外开放。举办好中国国际马戏节、WTA超级精英赛、2018年亚洲帆板锦标赛等活动。

　　扎实推进十件民生实事。一是疏导重点交通拥堵路段。改造提升屏东四路、沿河路交情侣路交叉口、梅界路交敬业路交叉口等10处拥堵点。建设一批人行过街设施。提高上下班高峰期和重大节假日路面见警率，重点疏导上冲检查站、珠海大道、金凤路、旅游路、柠溪路、桂花路等拥堵路口路段。二是大力发展公交事业。完成753台传统能源公交车更新工作，新增投放400台纯电动公交车。优化公交线网，新增10条以上公交线路，推广产业园区“微循环公交”。粤海路、桂花路、翠前路等道路新建公交专用道。三是加大幼儿园学位供给。新建、移交、收购8所公办幼儿园，其中建成3所、移交2所、收购1所、开工2所。实现全市普惠性幼儿园覆盖率达80%以上。四是提升道路保洁水平。定时对道路清洗和洒水防尘，城区主干道实行16小时保洁制，相应提高次干道和背街小巷保洁水平。五是推进保障性安居工程建设。加快建设南屏保障房项目，基本建成保障性住房1140套。新开工建设棚户区改造安置住房1600套以上。六是提高医疗卫生保障水平。基本公共卫生服务经费标准提高到每人每年60元以上。推进市妇幼保健院异地新建、市慢性病防治中心、西部医疗中心等项目建设。建成5个健康镇（街）、50个健康村（居）、100个健康“细胞”单元。七是推广使用管道天然气。新建40公里市政燃气管道，完成7.6万户老旧小区住宅的公共燃气管道加建工作。八是全面提升防灾减灾救灾能力。投入2亿元加强人、财、物保障，建设风暴潮灾害预报预警项目，高标准配备森林、高层建筑灭火救援装备，提升应急救援能力。九是开展污水整治行动。完成排水管网普查工作，对症下药，12条黑臭水体实现不黑不臭。十是改造提升老旧社区。启动翠香、东风、北堤等13个老旧社区环境改善行动，改善市政线网、公共照明、人行道、停车设施、消防通道等配套设施。

　　（八）以群众所需为施政所向，建设人民满意政府

　　建设法治政府。落实法治政府建设实施方案，完善行政执法体制机制，加大政务公开力度。深入推进政府法律顾问服务示范市建设。建立推动高质量发展的绩效评价机制。加大全民普法力度，推进公共法律服务体系建设。认真落实市人大及其常委会的各项决定，自觉接受市人大、市政协和社会各界的监督，办理好人大议案、建议和政协提案。

　　建设高效政府。增强干部队伍八种本领，知责有为，克服“慢、拖、虚”，以真抓的实劲、敢抓的狠劲、善抓的巧劲、常抓的韧劲，抓好各项工作落实。深化“一门式一网式”服务模式改革，践行“马上就办”的工作作风，推行“最多跑一次”服务，加强12345市民服务热线建设，让群众和企业切实感受到政府服务效率的提高。

　　建设廉洁政府。开展“不忘初心、牢记使命”主题教育。落实中央八项规定及实施细则精神，知止有戒。严守政治纪律和政治规矩，全面彻底肃清李嘉、万庆良流毒影响，持之以恒正风肃纪，保持惩治腐败高压态势。对财政资金分配、国有资产、政府投资等重点部门和岗位，强化内部流程监控，加强廉政风险防控和审计监督。构建“亲”“清”新型政商关系，营造风清气正的良好政治生态。

　　各位代表，新时代开启新征程，新使命呼唤新作为。我们将在市委的坚强领导下，在市人大和市政协的监督支持下，坚定信心、强化担当、狠抓落实，以时不我待、只争朝夕的精神投入工作，确保圆满完成今年的各项目标任务，以实实在在的工作成效向全市人民交上一份满意的答卷！
